# The Fifth International Neonatal and Maternal Immunization Symposium (INMIS 2019): Securing Protection for the Next Generation

**DOI:** 10.1128/mSphere.00862-20

**Published:** 2021-01-27

**Authors:** Manish Sadarangani, Tobias Kollmann, Gordean Bjornson, Paul Heath, Ed Clarke, Arnaud Marchant, Ofer Levy, Elke Leuridan, Rolando Ulloa-Gutierrez, Clare L. Cutland, Beate Kampmann, Surasith Chaithongwongwatthana, Ener Dinleyici, Pierre van Damme, Flor M. Munoz

**Affiliations:** a Vaccine Evaluation Center, BC Children’s Hospital Research Institute, Vancouver, British Columbia, Canada; b Division of Infectious Diseases, Department of Pediatrics, University of British Columbia, Vancouver, British Columbia, Canada; c Telethon Kids Institute, Perth Children's Hospital, University of Western Perth, Perth, Australia; d St. George’s University of London, London, United Kingdom; e Vaccines & Immunity Theme, Medical Research Council Unit, The Gambia, London School of Hygiene and Tropical Medicine (MRCG at LSHTM), Banjul, The Gambia; f Institute for Medical Immunology, Université Libre de Bruxelles, Charleroi, Belgium; g Precision Vaccines Program, Division of Infectious Diseases, Boston Children’s Hospital, Boston, Massachusetts, USA; h Harvard Medical School, Boston, Massachusetts, USA; i Broad Institute of MIT & Harvard, Cambridge, Massachusetts, USA; j Centre for the Evaluation of Vaccination, Vaccine & Infectious Disease Institute, University of Antwerp, Antwerp, Belgium; k Hospital Nacional de Niños Dr. Carlos Sáenz Herrera, Centro de Ciencias Médicas C.C.S.S., San José, Costa Rica; l African Leadership in Vaccinology Expertise (ALIVE), Faculty of Health Sciences, University of the Witwatersrand, Johannesburg, South Africa; m The Vaccine Centre, Clinical Research Department, London School of Hygiene and Tropical Medicine, London, United Kingdom; n Division of Infectious Disease in Gynecology and Obstetrics (InDiGO), Faculty of Medicine, Chulalongkorn University, Bangkok, Thailand; o Eskisehir Osmangazi University, Faculty of Medicine, Eskisehir, Turkey; p Department of Pediatrics, Baylor College of Medicine, Houston, Texas, USA; q Department of Molecular Virology and Microbiology, Baylor College of Medicine, Houston, Texas, USA; University of Maryland School of Medicine

**Keywords:** maternal immunization, neonatal, infant, vaccines in pregnancy, vaccination, immunology, integration, implementation, vaccine safety, vaccine acceptance

## Abstract

Despite significant progress in reaching some milestones of the United Nations Sustainable Development Goals, neonatal and early infant morbidity and mortality remain high, and maternal health remains suboptimal in many countries. Novel and improved preventative strategies with the potential to benefit pregnant women and their infants are needed, with maternal and neonatal immunization representing effective approaches. Experts from immunology, vaccinology, infectious diseases, clinicians, industry, public health, and vaccine-related social sciences convened at the 5th International Neonatal and Maternal Immunization Symposium (INMIS) in Vancouver, Canada, from 15 to 17 September 2019. We critically evaluated the lessons learned from recent clinical studies, presented cutting-edge scientific progress in maternal and neonatal immunology and vaccine development, and discussed maternal and neonatal immunization in the broader context of infectious disease epidemiology and public health. Focusing on practical aspects of research and implementation, we also discussed the safety, awareness, and perception of maternal immunization as an existing strategy to address the need to improve maternal and neonatal health worldwide. The symposium provided a comprehensive scientific and practical primer as well as an update for all those with an interest in maternal and neonatal infection, immunity, and vaccination. The summary presented here provides an update of the current status of progress in maternal and neonatal immunization.

## Abstract (precis)

Despite significant progress in reaching some milestones of the United Nations Sustainable Development Goals, neonatal and early infant morbidity and mortality remain high, and maternal health remains suboptimal in many countries. Novel and improved preventative strategies with the potential to benefit pregnant women and their infants are needed, with maternal and neonatal immunization representing effective approaches.

Maternal and pediatric morbidity and mortality remain at the forefront of the international public health agenda. Vaccines are safe and highly effective at reducing death and disability in young children, but most vaccines are given weeks to months after birth, while the highest pediatric mortality occurs around the time of birth and, specifically, within the neonatal period (the first 28 days) (1). Neonatal mortality now contributes nearly 50% to the overall global mortality of children under the age of 5 years, and novel or improved interventions are needed if this figure is to change. Vaccines, given either to the infant at birth to induce active immunity or to mothers during pregnancy to maximize the transfer of passive immunity via protective antibody (Ab) across the placenta or into breast milk, can play a key role in this context or provide other protective immune functions (1). The safest and most effective strategies for neonatal and maternal immunization need to be continuously reviewed. There is also a need for a better understanding of how to ensure that existing vaccines can reach vulnerable populations and to address the hesitancy about vaccine use, particularly during pregnancy. This is particularly relevant as we face an ongoing threat with the new coronavirus disease 2019 (COVID-19) pandemic. Many stakeholders, including researchers, policymakers, research funders, nongovernmental organizations, vaccine manufacturers, and frontline immunization providers, have to be involved to guarantee optimal and rapid translation of research findings and implementation of the best preventive strategies.

The 5th International Neonatal and Maternal Immunization Symposium (INMIS) convened experts from immunology, infectious diseases, vaccinology, clinicians, industry, public health experts, and social scientists in Vancouver, Canada, from 15 to 17 September 2019 to review the most relevant advances in maternal and neonatal immunization. The overarching focus of the conference was to review how best to secure protection for the next generation (i.e., from mother to infant) against potentially preventable infectious diseases via maternal and early-life immunization strategies. Over 250 participants attended the 2.5-day meeting that included 11 invited expert presentations, 28 oral presentations from submitted abstracts, 101 poster presentations, and 3 expert panel discussions. The meeting sessions were organized to begin with an opening overview keynote address. On the first day, there were sessions on the themes “Protecting newborns and infants through maternal immunization” and a “Multi-disciplinary approach to improve maternal vaccine uptake,” including a panel discussion, “Overcoming hurdles to increase maternal vaccination uptake.” The second day was dedicated to the themes “The mechanistic underpinnings of maternal and neonatal immunization” and “Promoting healthy infant life through optimizing neonatal immunization,” with the former including a panel and audience discussion, “How does the maternal-newborn immune dyad communicate?” Finally, the third day focused on the theme “The next generation of neonatal and maternal immunization research” with an audience discussion of “Is the field on the right path? What are we missing?” The closing keynote speech addressed the controversial issue of the ethics of maternal immunization research and implementation. A brief summary of the keynote presentations follows, with subsequent sections each dedicated to one of the themes of the symposium. In light of the current COVID-19 pandemic, the current state of knowledge on the impact of severe acute respiratory syndrome coronavirus 2 (SARS-CoV-2) on the maternal-infant dyad is briefly discussed to consider the output of the meeting in the perspective of the ongoing pandemic and given implications for SARS-CoV-2 vaccine development and the inclusion of special populations, such as pregnant women.

Shabir A. Madhi (University of Witwatersrand, South Africa) highlighted that despite global progress in reducing under-5-year-old childhood deaths as part of the Millennium Development Goals agenda (1990 to 2015), reductions in mortality rates during the neonatal period lagged behind those for children 1 to 59 months of age. Furthermore, there has been limited focus on the prevention of stillbirths, despite the stillbirth rate exceeding neonatal mortality rate in many low- and middle-income countries (LMICs). United Nations Sustainable Development Goal (SDG) 3.2 aspires to prevent all preventable deaths of newborns and children by 2030, as well as to reduce neonatal mortality to 12 per 1,000 live births. In addition, the Every Newborn Action Plan aims to reduce stillbirth rates from 20 to 10 per 1,000 births by 2035.

Dr. Madhi emphasized that quantifying the potential of maternal vaccines, or other prophylactic interventions, in reducing maternal morbidity, stillbirth rates, and early-infancy deaths requires in-depth and objective assessment of the causes of newborn and infant deaths. Recent surveillance using minimally invasive tissue sampling has unmasked the hitherto largely neglected contribution of infection as the dominant immediate cause of stillbirth and neonatal deaths, including deaths usually attributed to premature birth. This approach also has the potential to identify the potential contribution of pathogens for which there are no current vaccines, for example, group B *Streptococcus* (GBS) and Gram-negative bacteria. Such data are essential to inform the prioritization of research and interventions aimed at reducing maternal and childhood deaths.

The potential of maternal immunization (coupled with other possible changes) in reducing neonatal mortality is manifest by the near elimination of neonatal tetanus globally following the recommendation of routine tetanus vaccine immunization of pregnant women (2). Furthermore, since the 2009 H1N1 influenza pandemic, there has been an acceleration of research on the potential impact of maternal immunization in improving maternal and child health, including the demonstrated effectiveness of acellular pertussis and influenza vaccination of pregnant women in reducing severe pertussis during early infancy and seasonal influenza illness in the women and their young infants, respectively. Pertussis and influenza are important causes of pneumonia in young infants, and pneumonia is a prominent cause of death in early life (3). The first efficacy trial of a respiratory syncytial virus (RSV) vaccine in pregnant women demonstrated its potential for preventing the leading cause of severe lower respiratory tract infection during early infancy, particularly in LMICs (4). The prevention of RSV lower respiratory tract illness in early infancy by immunization in pregnancy may also be augmented through the administration to infants of new-generation monoclonal antibodies that have an extended half-life (5).

In recent years, outbreaks of the H1N1 pandemic influenza, Zika, and Ebola viruses have severely and uniquely affected pregnant women and their offspring. Ruth Karron (Johns Hopkins Bloomberg School of Public Health, USA) underlined that pregnant women must be proactively considered in research agendas and in efforts to deploy vaccines against emerging infectious diseases; these vaccines have rarely been designed or developed with pregnant women in mind. For this and other reasons, pregnant women have in some cases been denied vaccines that would have protected them and their offspring from severe epidemic threats. This has become particularly relevant now amid the COVID-19 pandemic. Recommendations to guide the development and deployment of appropriate vaccines in these situations did not exist prior to the recent Ebola outbreak. To address this need, the Pregnancy Research Ethics for Vaccines, Epidemics, and New Technologies (PREVENT) Working Group was formed. This is a multidisciplinary, international team of 17 experts specializing in bioethics, maternal immunization, maternal-fetal medicine, obstetrics, pediatrics, philosophy, public health, and vaccine research and policy. After consultation with >100 additional experts in ethics, public health, vaccine science, maternal and child health, and regulatory affairs, the group developed a guidance document that put forth 22 recommendations across the domains of epidemic preparedness, vaccine research and development, and vaccine deployment. A key recommendation was the presumption of inclusion, suggesting a change to the traditionally default position, so that pregnant women would be included in vaccine development and deployment unless their exclusion can be justified from a scientific and ethical standpoint. The presumption of inclusion reframes decisions about investments in vaccine research, development, and delivery in ways that are profoundly important for public health and equity, and the principles are similarly applicable to neonates. The PREVENT Working Group recommendations and framework will be useful when weighing the potential risks and benefits for pregnant women and their newborns in the development and deployment of vaccines against current and emerging epidemic threats, such as COVID-19. Many of the PREVENT recommendations may also be relevant as the inclusion of pregnant women is more broadly considered in the context of biomedical research.

## PROTECTING NEWBORNS AND INFANTS THROUGH MATERNAL IMMUNIZATION

Data were presented on the various challenges and opportunities related to implementation of maternal immunization programs in different settings, including LMICs and high-income countries (HICs). Philipp Lambach (World Health Organization [WHO], Switzerland) highlighted the efforts by the WHO to support maternal immunization platforms in LMICs, using tetanus vaccination in pregnancy as a proof of concept of this approach. The Maternal Immunization and Antenatal Care Situation Analysis (MIACSA) project, a collaborative effort between the WHO Departments of Immunization, Vaccines, and Biologicals (IVB) and Maternal, Newborn, Child, and Adolescent Health (MCA), was undertaken in 2016 to 2019 (6, 7). MIACSA assessed maternal tetanus vaccine service delivery strategies and factors associated with effective delivery in low-resource settings, including collaborations between the immunization program and antenatal care clinic programs, human resources, finances, information systems, and surveillance. These are key issues that need to be considered in LMICs to prepare countries for future maternal immunization introduction, including vaccines that are required for existing and emerging pathogens. Marta Nunes (University of the Witwatersrand, South Africa) reported on how maternal immunization programs can be effectively implemented in LMICs, using the example of influenza vaccination campaigns targeted at pregnant women at antenatal clinics in South Africa. Annette Regan’s (Texas A&M University, USA) findings from the PREVENT network project emphasized the potential impact of influenza infection on pregnancy and the newborn (8). Although influenza infection during pregnancy was not often diagnosed in this international cohort, acute influenza infection during pregnancy was associated with an increased risk of preterm birth and low birth weight. To highlight the challenges in vaccine safety surveillance in these settings, Ed Clarke (MRC Unit, The Gambia) reported on the impact of using the Global Alignment of Immunization Safety Assessment in Pregnancy (GAIA) Consortium case definitions on the reporting of preterm birth in a maternal vaccination trial in The Gambia, comparing levels of variability in the accuracy of gestational age estimates based on either symphysis-fundal height or reported last menstrual period data.

Deshayne Fell (University of Ottawa, Canada) described how electronic health care data, such as insurance claims, health administrative databases, registries, and electronic health records, are being increasingly used to address important research on vaccine coverage and safety and the effectiveness of maternal immunization in HICs. In the United States, studies using the Vaccine Safety Datalink (VSD) have shown the safety of influenza immunization during pregnancy, with no increases in risks of proximal adverse events in pregnant individuals or specific adverse obstetric events, such as hyperemesis, gestational hypertension, gestational diabetes, preeclampsia, or chorioamnionitis (9, 10). Similar studies in Norway, Denmark, and Canada have reported no association between pandemic influenza vaccine and fetal, neonatal, or longer-term (to age 5 years) pediatric outcomes (11–14). Other studies from the United Kingdom and United States have confirmed the safety of pertussis immunization during pregnancy (15, 16). Meghan Laverty (University of Ottawa, Canada) reported on a population-based data linkage study from Canada, identifying no association between exposure to the tetanus, diphtheria, and pertussis (Tdap) vaccine during pregnancy and early-childhood health outcomes. Annette Regan highlighted the opportunity of data linkage studies to be used internationally. In the study that she described, a retrospective cohort of pregnant women in four high-income countries was established using administrative health data. Hospital records were combined with laboratory testing data and birth records to identify acute respiratory or febrile illness-related admissions during pregnancy and infant outcomes of preterm birth and low birthweight. Such studies demonstrate the potential perinatal benefits of preventing maternal severe respiratory disease, such as via influenza vaccination (8). Different approaches have successfully been used for these data linkage studies, including (i) a distributed data model in which autonomous software on a centralized secure server accesses site-specific patient-level data to enable a single combined analysis and (ii) a common data model in which there is harmonization of protocols to standardize variable definitions and statistical analysis, with separate site-specific analyses enabling final meta-analysis of site-specific estimates (17, 18). A number of challenges are encountered using these approaches, including bias due to differential health care-seeking behaviors, incomplete documentation, miscoding by medical records staff, and incomplete linkage or limited clinical or demographic information.

## MULTIDISCIPLINARY APPROACH TO IMPROVE MATERNAL VACCINE UPTAKE

Rolando Ulloa-Gutierrez (Hospital Nacional de Niños, Costa Rica) illustrated how researchers and governments need to collaborate closely in the field of maternal and neonatal immunization to ensure that research findings are translated into policy. In 2017, the Pan American Health Organization (PAHO) and WHO published the *Maternal and Neonatal Immunization Field Guide for Latin America and the Caribbean* (19), positioning maternal and neonatal immunization at the highest political level and as a commitment for member states. Multiple challenges remain, including achieving high vaccine coverage, protecting dedicated government funding for immunization programs, and reducing inequality in access to health services. While strong evidence exists for political authorities to include maternal immunization as a cost-effective health care priority, catalysts for translating this into policy in Latin America have been political commitment, endorsement by scientific societies, an established “culture of vaccination,” widespread access to antenatal care, and context-specific communications. Facilitators of immunization programs relate to the potential for cost savings, improved population health, and higher economic productivity. Immunization success stories, such as the formal elimination of measles, rubella, and congenital rubella syndrome in the region, have been a key facilitator to ensure ongoing engagement. When solid immunization systems are not in place, global events, such as the COVID-19 pandemic, can leave many countries highly vulnerable, since the disruption to immunization services can lead to a reemergence of vaccine-preventable diseases, such as measles (20).

Alba Vilajeliu (PAHO, USA) presented data from a study of the current state of maternal and neonatal immunization policies, strategies, and practices in Latin America and of the knowledge and perceptions of pregnant women and health workers in this regard, including the importance of integration between immunization and antenatal care services, similar to the goals of MIACSA. The study suggested an important role of health care provider (HCP) recommendations. Penda Johm (MRC Unit, The Gambia) reported that high levels of acceptance of maternal immunization in The Gambia are based on previous vaccination experiences, sensitization messages from trusted HCPs, and monetary incentives. Further data reinforcing the importance of an HCP recommendation was provided by Neisha Sundaram (London School of Hygiene and Tropical Medicine [LSHTM], UK); recommendations from doctors were highly valued by pregnant women in Bengaluru, India. However, awareness of and access to vaccines other than that for tetanus were limited, highlighting the need for better information about maternal vaccines. Karina Top (Canadian Center for Vaccinology, Canada) presented data from an HIC setting, further examining the information provided about vaccines given to pregnant women in product monographs and demonstrating that clear, evidence-based product monographs could support increased vaccine uptake in pregnancy (21). Eliz Kilich (LSHTM, UK) provided data from a systematic review and meta-analysis to examine the factors influencing vaccination decision-making among pregnant women, which further reinforced the findings reported from the various studies mentioned above; interventions designed to facilitate HCP recommendations are likely to have the strongest impact on maternal vaccine uptake (22). Using a health beliefs model which included factors such as disease susceptibility, severity, HCP recommendations, benefit, or utility, disease susceptibility was not a significant factor for vaccine acceptance in this study. Vaccine safety, however, was a key determinant, and conditions such as autism, narcolepsy, miscarriage, and infertility were noted concerns.

## THE MECHANISTIC UNDERPINNINGS OF MATERNAL AND NEONATAL IMMUNIZATION

A session on the mechanistic underpinnings of maternal and neonatal immunization was organized to (i) illustrate the potential of systems immunology for the understanding of the immunobiology of the mother-infant dyad, (ii) provide an update on the immunobiology of pregnancy and its relevance for vaccine responses, and (iii) communicate new findings on the rules and mechanisms underlying transplacental transfer of maternal antibodies. A successful pregnancy requires dynamic changes in the maternal and fetal immune systems. After birth, the immune systems of the newborn and young infant develop to meet the challenges of tolerance to commensals and immunity to infectious pathogens (23). The impacts of these dynamic changes on the quality of immune responses during pregnancy and soon after birth remain poorly understood. The emergence of new technologies and computational tools allowing the integration of large and complex data sets opens new avenues for understanding the immunobiology of the mother-infant dyad (1).

John Tsang (National Institutes of Health, USA) discussed the potential of systems biology to discover new components regulating immune responses in pregnancy and infancy and to develop quantitative models of how these components interact at the level of the mother-infant dyad. Systems biology approaches have been successfully applied to the analysis of immune responses to influenza immunization in healthy adults. Baseline (preimmunization) and vaccine-induced parameters, including blood cell populations, genes, and serum proteins, are predictors of influenza vaccine responses (24). Dr. Tsang discussed changes in transcriptional profiles of peripheral blood immune cells following pertussis immunization of pregnant women and young infants that are candidate biomarkers for predicting vaccine responses. Beyond antibodies, correlations were observed between maternal and infant serum protein profiles that may represent important maternal determinants of infant immunity. Single-cell transcriptome analyses identified key myeloid cell populations driving dynamic changes of immune states during the first days of life. Together, these data demonstrated the very exciting potential of systems biology to inform the development of optimized maternal and infant vaccination strategies.

Transfer of maternal antibodies across the placenta provides rapid protection to the infant against pathogens to which the mother is immune. Maternal antibodies of different specificities are not transferred equally, but the rules underlying variability in the transfer process remain poorly understood (25). Madeleine Jennewein (Ragon Institute, USA) reported on the use of a systems serology approach to investigate the glycosylation profile and the functional properties of the crystallizable (Fc) fragment of IgG. Newborn cord blood contained higher concentrations of maternal antibodies promoting NK cell degranulation than maternal blood (26). Data modeling and Ab glycoengineering experiments indicated that this preferential transfer of NK cell-activating Abs was dependent on the expression of galactose by IgG Fc. These data demonstrated that the transfer of maternal antibodies across the placenta selects specific structural and functional features that may be critical for optimal neonatal immunity. A systems serology approach was used by Marcela Pasetti (University of Maryland, USA) to study the impact of pregnancy on antibody responses to maternal immunization against influenza; two clearly distinct immune profiles were identified in pregnant and nonpregnant women in response to seasonal influenza vaccines. Pregnancy had been associated with increased galactosylation of total IgG, but its impact on vaccine-induced antibodies is unknown. Sepideh Dolatshahi (Ragon Institute, USA) described a systems serology approach to analyze the evolution of maternal antibody features over the first months of life in preterm versus term newborns, revealing significant differences in maternal IgG1 and binding to FcgR. Maternal antibody features of the two groups then converged during the first months of life, in parallel with their decay, as observed at the level of serum proteome and immune cell phenotypes (27).

When transferred at high concentrations, maternal antibodies can modulate infant vaccine responses and commonly decrease their magnitude. However, the impact of maternal antibodies on the functional properties of vaccine-induced antibodies remains largely unexplored. Nasamon Wanlapakorn (Chulalonglorn University, Thailand) presented the results of a randomized trial evaluating the functional properties of antibodies induced by acellular pertussis (aP) versus whole-cell pertussis (wP) vaccines in infants born to mothers who received the Tdap vaccine during pregnancy (28). The study showed that in infants, the bactericidal activity of pertussis antibodies was highly dependent on the presence of complement *in vitro* and that wP vaccination induced higher serum bactericidal activity than aP vaccination. Although maternal immunization was associated with lower titers of infant IgG versus pertussis antigens, its impact on serum bactericidal activity was limited, suggesting that high-quality infant antibodies may be produced even under the cover of maternal antibodies. Studies also suggested that maternal antibodies have a limited impact on infant T cell responses to vaccines (29). Marjolein Orije (University of Antwerp, Belgium) reported a study of T cell responses to pertussis vaccination in preterm and term infants, suggesting that maternal pertussis vaccination did not influence these cell-mediated responses (29).

The panel discussion of the session emphasized some of the challenges of applying systems biology to the analysis of vaccine responses in pregnant women and infants, including uncertainties about optimal sample size and limitations in the number of samples that can be collected from pregnant women and infants. Systems biology may inform personalized medicine, but its relevance for global health remains to be convincingly demonstrated. Systems biology may inform vaccine discovery and development to optimize safety and efficacy, but the path to achieve this remains to be delineated. To provide mechanistic insights, clinical systems biology studies should integrate upstream and downstream approaches, including animal models and human *in vitro* models, to define mechanisms of action (30).

## PROMOTING HEALTHY INFANT LIFE THROUGH OPTIMIZING NEONATAL IMMUNIZATION

David Goldblatt (University College London, UK) highlighted the global variation in the etiology of community-acquired neonatal sepsis (31) and suggested that country-level vaccination strategies should be prioritized according to this epidemiology. Major challenges for neonatal immunization include perceptions regarding safety and differences in neonatal immunologic responses from those of older children and adults (32). Although much of the information about vaccine coverage rates in Africa are available for children during the first year of life, there is little information about timely administration of vaccines given at birth (hepatitis B and Mycobacterium bovis BCG). A publication from The Gambia (33) revealed that although 93% of 10,851 children did receive the first dose of the hepatitis B vaccine by the age of 6 months, only 1% were vaccinated at birth, 5% by day 7, and 58% by day 28. Therefore, interventions to ensure the successful and timely implementation of neonatal immunization policies are needed.

BCG remains a significant interest because of the potential for nonspecific (heterologous) immunomodulatory effects resulting in protection against a range of infections, as postulated for COVID-19 (34–36). However, the duration, magnitude, and importance of these heterologous effects remain undetermined at this time, and further studies are ongoing (37). Byron Brook (University of British Columbia [UBC], Canada) presented a study which characterized the mechanism underlying BCG-induced heterologous immunity against polymicrobial sepsis in neonatal mice and humans (38). Neutrophils seemed to be the most important factor; BCG vaccination led to rapid granulopoiesis and ultimately protection from polymicrobial sepsis within 3 days of vaccination. An additional challenge is the fact that BCG vaccine production is not standardized. Kinga Smolen (Boston Children’s Hospital, USA) compared licensed BCG vaccines, demonstrating marked differences in their contents of live mycobacteria, which possibly contribute to distinct cytokine and chemokine induction *in vitro*. It is not currently known which BCG substrain/formulation offers the best protection.

Paul Heath (St. George’s, University of London, UK) highlighted that preterm infants have lower-than-normal concentrations of maternal IgG, resulting in increased susceptibility to infection (39), including pertussis, pneumococcus, rotavirus, influenza, and RSV. This is in part also due to reduced cellular immune responses and lower lymphocyte counts (40), as well as lower levels of maternal antibodies. Preterm infants are often excluded from prelicensure studies of new vaccines, efficacy studies are almost nonexistent, and immunogenicity studies include small numbers, different schedules, and different populations and have varied inclusion and exclusion criteria, creating a challenge in evidence-based decision-making for this population. Preterm infants usually have lower antibody concentrations after primary vaccination than full-term infants, but proportions achieving protective concentrations may be equivalent for vaccines for which correlates of protection have been described (41). Booster doses are particularly important in these infants. A prospective study in The Netherlands (42) showed that immunizations were often delayed in preterm infants, being lowest (37%) in infants <28 weeks of gestational age. Potential adverse events following immunization (AEFIs), such as apnea and major cardiorespiratory events, occur more frequently in preterm infants than in term infants, but overall vaccines are safe in preterm infants, who should be immunized according to their chronological age and not their corrected gestational age (43). In hospitalized preterm infants, the risk of cardiorespiratory events after immunization is strongly related to the presence of prematurity-associated adverse events in the 24 h preceding immunization but not to gestational age, birth weight, or postnatal age. Maternal immunization is a key mechanism by which these highly vulnerable infants may be protected, provided that vaccination occurs in the second trimester and significant antibody transfer is achieved prior to delivery, although even then, this would leave the extremely preterm infants vulnerable. For example, infants born to mothers vaccinated with the Tdap vaccine during pregnancy had significantly higher antibody concentrations at 2 months for all vaccine antigens than infants of unvaccinated mothers (44).

Rebecca Ford (Papua New Guinea Institute of Medical Research, Papua New Guinea) explored approaches to protect infants in Papua New Guinea against pneumococcal disease through different vaccination schedules, including neonatal immunization with a pneumococcal conjugate vaccine (PCV), and suggested that there may be a benefit to a pneumococcal polysaccharide vaccine booster dose in such a strategy. Results of a randomized controlled trial in The Netherlands on modulation of infant immune responses after maternal Tdap vaccination were complemented with the opsonizing capacities of the elicited antibodies in infants. The opsonizing capacity of antibodies may help us to understand whether this modulation is clinically relevant. The opsonizing capacity was higher in the offspring of women receiving the Tdap vaccine before primary infant vaccination and lower after priming, with similar findings before and after the second-year-of-life booster. Differences resolved at 2 years of age.

Bahaa Abu Raya (UBC, Canada) reported a meta-analysis of most of the globally available data on infant immune responses to pertussis, tetanus, diphtheria, and pneumococcal vaccines after maternal Tdap vaccination. Although schedules, epidemiologies, and vaccines used differed among countries and trials, the modulatory effect of the Tdap vaccine in pregnancy was observed in most studies for all vaccine antigens.

Lieke van den Elsen (University of Western Australia, Australia) presented data on the detection of malaria antigens in breastmilk and suggested that such antigens may promote immune defenses against Plasmodium falciparum, leading to direct infant immunization and reduced malaria risk in breastfed infants.

## THE NEXT GENERATION OF NEONATAL AND MATERNAL IMMUNIZATION RESEARCH

Studies conducted in Southeast Asia and sub-Saharan Africa (SSA) have highlighted different contributions of various pathogens to neonatal sepsis, necessitating the development of new vaccines for maternal and neonatal immunization (45–47). Stephanie Schrag (Centers for Disease Control and Prevention, USA) gave a comprehensive overview of research needs for the next generation of maternal and neonatal vaccines, including vaccines against Zika virus (ZIKV), cytomegalovirus (CMV), malaria, and GBS. Severe congenital microcephaly associated with ZIKV disease in pregnant women spurred rapid vaccine development during the outbreak in 2015. Despite the apparent resolution of the epidemic, several agencies are continuing ZIKV vaccine development to generate a safe vaccine available for future outbreaks (48). CMV causes mild infections in healthy adults; however, rare but severe complications are observed in 4% to 6% of congenitally infected infants. Identification of the most suitable target population for vaccination against CMV is challenging. Vaccine development is advanced, but no vaccines against CMV are currently licensed (49). Pregnant women are at increased susceptibility for malaria-related disease, complications, and death, and maternal immunization should be considered during reviews of phase IV safety data and for future trials of the PfSPZ malaria vaccine, which is in phase 2 testing (50). A monovalent pertussis vaccine may be used in maternal and birth dosing regimens, be more affordable than the Tdap vaccine, and reduce the immune modulation in infants described above (51). GBS is a leading cause of neonatal sepsis (52). Early-onset disease (<7 days) can be prevented by intrapartum antibiotic prophylaxis (IAP) (53); however, implementation of IAP is not desirable or feasible in many areas due to antimicrobial resistance (AMR) concerns or resource constraints. Several GBS vaccines are in preclinical and clinical development; however, challenges exist, as large trials will need to be conducted in areas with adequate disease burdens, established disease surveillance programs, clinical trial experience, and the capacity to identify adverse events (54).

Kimberly Center (Pfizer Inc., USA) presented results of a first-in-human study of a hexavalent GBS conjugate vaccine (GBS6) (NCT03170609). Healthy adults were enrolled into a randomized, placebo-controlled trial of a single dose of hexavalent GBS vaccine containing 5, 10, or 20 μg capsular polysaccharide of serotypes Ia, Ib, II, III, IV, and V, with or without aluminum phosphate (AIPO_4_). Mild-to-moderate local and systemic reactions were common, but none led to withdrawal from the trial. The vaccine yielded a robust antibody response, supporting progression to clinical trials in pregnant women.

AMR has been listed as one of the top 10 global threats to human health in 2019, with deaths related to AMR estimated to be ∼700,000 annually and projected to increase to 10 million by 2050 (55). A recent systematic review (47) reported that AMR in neonatal sepsis in SSA increased in 2008 to 2018 compared to that in 1980 to 2007. Staphylococcus aureus contributed to 25% of neonatal sepsis cases in both time periods. Klebsiella pneumoniae and Escherichia coli were the most commonly identified Gram-negative bacteria. In the later time period studied, 68% and 27% of bacterial pathogens were resistant to beta-lactams and aminoglycosides, respectively (3). Padmini Srikantiah (Bill & Melinda Gates Foundation, USA) described that development of a K. pneumoniae vaccine for adults is under way and may be considered for vaccination of pregnant women to reduce neonatal sepsis in the future (56). A recombinant monoclonal antibody to staphylococcal protein A (SpA) has shown promising results in a mouse model and may be considered at birth (57).

Louis Fries (Novavax, Inc., USA) shared the results of a maternal immunization trial with a respiratory syncytial virus F protein vaccine (4). The vaccine was found to be safe, with no significant negative vaccine effects on pregnancy, delivery, or infant birth outcomes or adverse events. The overall vaccine efficacy was 39% against medically significant RSV lower respiratory tract infection, with increased efficacy for more-severe disease endpoints. Vaccine efficacy was higher in LMICs than in HICs, which may have been due to higher severe disease incidence, earlier immunization, better alignment with RSV season, and/or more persistent breastfeeding in LMICs. This vaccine has the potential to significantly impact clinical pneumonia in infants, especially in LMICs.

*Shigella* is an important cause of diarrhea in children; however, there is no licensed vaccine available yet. Esther Ndungo (University of Maryland School of Medicine, USA) presented the results of a 2-year longitudinal study of 100 mothers and infants conducted in Malawi to determine the repertoire, functional capacity, and maternal-infant transfer efficiency of antibodies against *Shigella*, identifying differences between levels of transfer of all *Shigella*-specific IgGs and functional antibodies; further understanding of this would be critical for any *Shigella* vaccine.

## COVID-19 AND THE MATERNAL-INFANT DYAD

During the current COVID-19 pandemic, concerns have been raised about the possibility of vertical or perinatal transmission of SARS-CoV-2 and the effect of the infection on the pregnant woman, the fetus, or the infant. Disease severity and complications of COVID-19 appear to be relatively low during pregnancy, although multiple international studies are ongoing (58). Multiple recent reports suggest an increased risk of intensive care unit admission, invasive ventilation, and possibly mortality in pregnant individuals compared with that for nonpregnant individuals of reproductive age (59–61). Considerations for inclusion of pregnant women in the evaluation of COVID-19 vaccines are already under way.

The most common clinical manifestations of COVID-19 in pregnancy have been reported as fever (40%) and cough (39%). Pregnant women are less likely to report fever and myalgia than nonpregnant women of reproductive age. Increased maternal age, high body mass index, chronic hypertension, and prior diabetes have been linked with severe COVID-19 in pregnancy. Regarding obstetric complications, several publications suggest the possibility of adverse obstetric outcomes in women with COVID-19, including Caesarean section, premature birth, low birth weight, and adverse pregnancy events (62, 63). However, rates vary by the location, type, and size of studies. Multiple registries are in place to continue to monitor this as the pandemic progresses, and carefully controlled analyses will be required to appropriately determine any modified risk due to COVID-19 in pregnancy. SARS-CoV-2 has not been detected in human milk in multiple studies, except for one case in Germany (64).

Vertical transmission of SARS-CoV-2 to the infant is a potential concern, for which initial case series did not show substantial evidence (65–67). Some recent reports suggest the probability of vertical transmission (68, 69). A case definition and potential mechanisms of vertical transmission have been suggested (69–71). A recent systematic review of neonatal COVID-19 infections identified that 71% were confirmed/probably acquired postnatally, 3.3% were acquired intrapartum (with an additional 14% probably/possibly acquired intrapartum), and 5.7% were confirmed congenital cases (with a further 6.5% probably/possible congenital) (68). Variations in testing strategies around the world have made it challenging to confirm accurately the true vertical transmission rates.

## CONCLUDING REMARKS

Maternal and neonatal immunization is an effective key strategy in reducing death and significant morbidity from infectious diseases globally. While significant progress has been made in the implementation of maternal immunization programs in various regions and settings worldwide, much remains to be done. The integration of maternal immunization and antenatal care programs is critical, and local solutions which can adapt to different specific needs and a variety of settings are required. Our increased understanding of the mechanistic underpinnings of maternal and neonatal immunization will enable further vaccine development to be based on a bedrock of scientific evidence. Ongoing surveillance of known and emerging infectious diseases affecting women during pregnancy and infants in early life is required to ensure that the development and implementation of safe new vaccines remain relevant to the prevention of severe disease in these potentially highly susceptible populations. At a time of global challenges to health care systems worldwide due to the SARS-CoV-2 pandemic, ensuring the continuation of vaccination programs for pregnant women and newborns must remain an international priority.
